# Mining of the Pyrrolamide Antibiotics Analogs in *Streptomyces netropsis* Reveals the Amidohydrolase-Dependent “Iterative Strategy” Underlying the Pyrrole Polymerization

**DOI:** 10.1371/journal.pone.0099077

**Published:** 2014-06-05

**Authors:** Chunlin Hao, Sheng Huang, Zixin Deng, Changming Zhao, Yi Yu

**Affiliations:** 1 Key Laboratory of Combinatory Biosynthesis and Drug Discovery, Ministry of Education, School of Pharmaceutical Sciences, Wuhan University, Wuhan, China; 2 State Key Laboratory of Bioorganic and Natural Products Chemistry, Shanghai Institute of Organic Chemistry, Chinese Academy of Sciences, Shanghai, China; 3 Hubei Engineering Laboratory for Synthetic Microbiology, Wuhan Institute of Biotechnology, Wuhan, China; University of New South Wales, Australia

## Abstract

In biosynthesis of natural products, potential intermediates or analogs of a particular compound in the crude extracts are commonly overlooked in routine assays due to their low concentration, limited structural information, or because of their insignificant bio-activities. This may lead into an incomplete and even an incorrect biosynthetic pathway for the target molecule. Here we applied multiple compound mining approaches, including genome scanning and precursor ion scan-directed mass spectrometry, to identify potential pyrrolamide compounds in the fermentation culture of *Streptomyces netropsis*. Several novel congocidine and distamycin analogs were thus detected and characterized. A more reasonable route for the biosynthesis of pyrrolamides was proposed based on the structures of these newly discovered compounds, as well as the functional characterization of several key biosynthetic genes of pyrrolamides. Collectively, our results implied an unusual “iterative strategy” underlying the pyrrole polymerization in the biosynthesis of pyrrolamide antibiotics.

## Introduction

Natural products (NPs) have been the major sources for clinical drug discovery and development for many decades [1], [2]. NPs with novel activities or skeletons are constantly needed to antagonize newly emerging threats to human health [3]. In recent years, the explosion of genome sequencing has led to rapid development of novel NP screening approaches which have greatly increased the number and diversity of NPs inventories [4]–[8]. As a correlation to this increase, understanding how the NPs are biosynthesized is also very important. Accessing to the mechanisms underlying NPs biosynthesis will not only improve our knowledge of various kinds of enzymatic reactions, but also pave the ways for future combinatorial biosynthesis which can guide medicinal chemistry in developing more applicable NP-derived drugs [9]. Adequate structural information of biosynthesis intermediates or analogs is generally required to establish an unambiguous biosynthetic pathway for a particular NP. However, due to the low concentrations or insignificant bio-activities of individual candidates or insufficient speculation on the candidates' structures, potential intermediates and analogs accumulated in the fermentation culture of the producing strain were often overlooked in liquid chromatography (LC) and mass spectrometry (MS) analysis. Therefore, the progress toward revealing the NPs' biosynthetic mechanisms has significantly lagged behind those toward NP discovery and screening, thus prompting the need for effective solutions [10]–[14].

**Figure 1:**
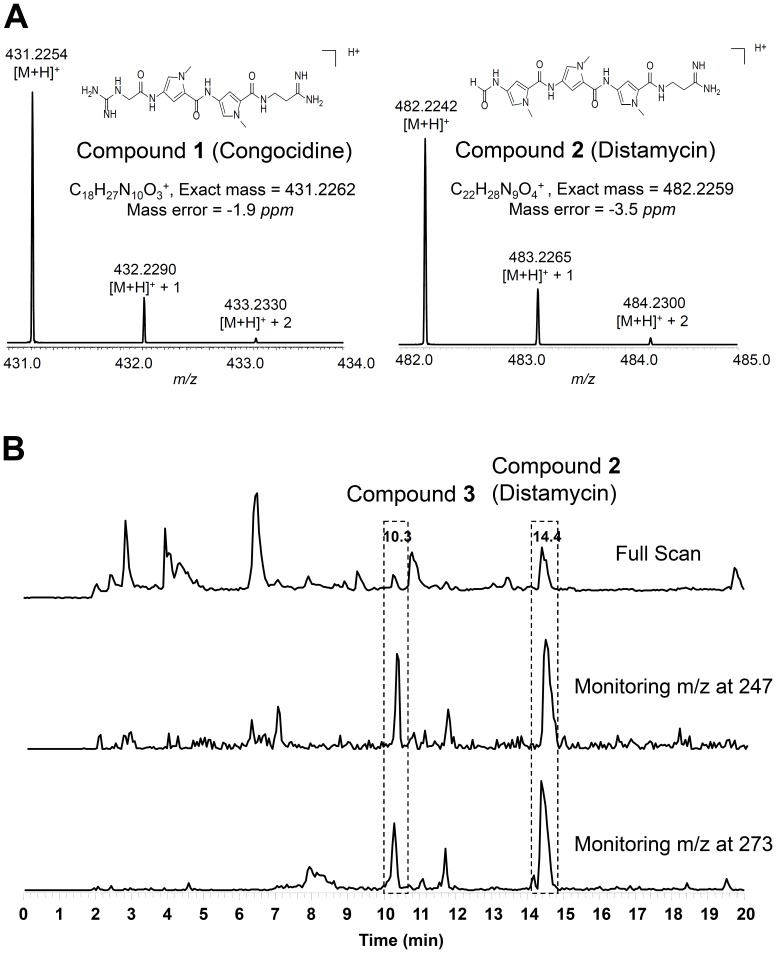
Identification of Congocidine (1), Distamycin (2), and a novel pyrrolamide compound (3) in *S. netropsis*. (A) High resolution mass spectrum of Congocidine and Distamycin. (B) Precursor ion scan-directed mass spectrum to identify compound **3**. Base peak chromatograms of precursor ion scan are shown. Ions of *m*/*z* 273 and 247 are daughter ions of compound **3**, and were used as the queries.

Pyrrolamides, biosynthesized by *Streptomyces* and related actinobacteria, are a class of poly-pyrrolic natural products containing one or more pyrrole-2-carboxamide moieties in their structures. Most pyrrolamides, including congocidine (compound **1**, Figure 1A), distamycin (compound **2**, Figure 1A), and pyrronamycin B, are found to possess the ability to bind to specific DNA sequences, which enables this compound group with many desirable biological activities (e.g., anti-virus, anti-bacteria and anti-tumor) [15]–[18]. Although the discovered natural pyrrolamides are still too toxic for clinical use, these molecules are still attractive in the field of pharmacology because their selective DNA sequence binding features may inspire the development of special drugs [19]–[22]. Additionally, numerous efforts have been made to chemically synthesize several DNA-binding agents based on pyrrolamide structures [23]. Thus, exploring novel NPs belonging to the pyrrolamide family can provide more skeleton hints to current DNA-binding pharmaceutical research. Recently, the first pyrrolamide gene cluster directing congocidine biosynthesis is identified in *Streptomyces ambofaciens* [24]. Juguet *et al*. demonstrated that congocidine is assembled by an iterative nonribosomal peptide synthetase (NRPS). In another work, nearly every gene in the congocidine gene cluster is separately inactivated, and LC-MS analysis of the related mutants showed that 4-acetamidopyrrole-2-carboxylate is the key precursor for pyrrolamide biosynthesis [25]. However, the main mechanism underlying the control of pyrrole polymerization, which may be the most intriguing question in oligo-pyrroles NP biosynthesis, has not yet been understood.

NPs sharing a common structure core can be fragmented in tandem MS to give characteristic daughter ions. Precursor Ion Scan (PIS), a MS scan mode that detects certain ions, has been applied to screen for compounds which probably belong to the same family. Some of the compounds identified in this way may be novel ones if their parent ions have a unique mass-to-charge (*m*/*z*) ratio readout. In this study, we identified six novel pyrrolamides from a single strain of *Streptomyces* by genome mining and PIS-directed mass spectrometry. The structure information of these compounds and the functional characterization of several key biosynthetic genes provided us important clues to solve the puzzle of pyrrole polymerization in pyrrolamides biosynthesis.

## Results and Discussion

### Discovery of novel pyrrolamide NPs in *Streptomyces netropsis*

Genome mining seeking for potential pyrrolamide producers was performed by using cgc2* as the gene bait, which was reported to confer resistance to congocidine on *S. ambofaciens* [24]. *S. netropsis* CGMCC 4.1650 was thus identified as a candidate among dozens of *Streptomyces* strains obtained from China General Microbiological Culture Collection. High resolution LC-ESIMS (HR-LC-ESIMS) analysis of the crude extract from this strain's fermentation culture revealed two major peaks with [M+H]^+^ ions at *m*/*z* 431.2254 and 482.2242 (Figure 1A). Further, tandem MS analysis of both ions' fragments and ^1^H NMR inspection confirmed that these two compounds are congocidine (compound **1**) and distamycin (compound **2**) (Figure S1 in File SI) [15].

**Figure 2:**
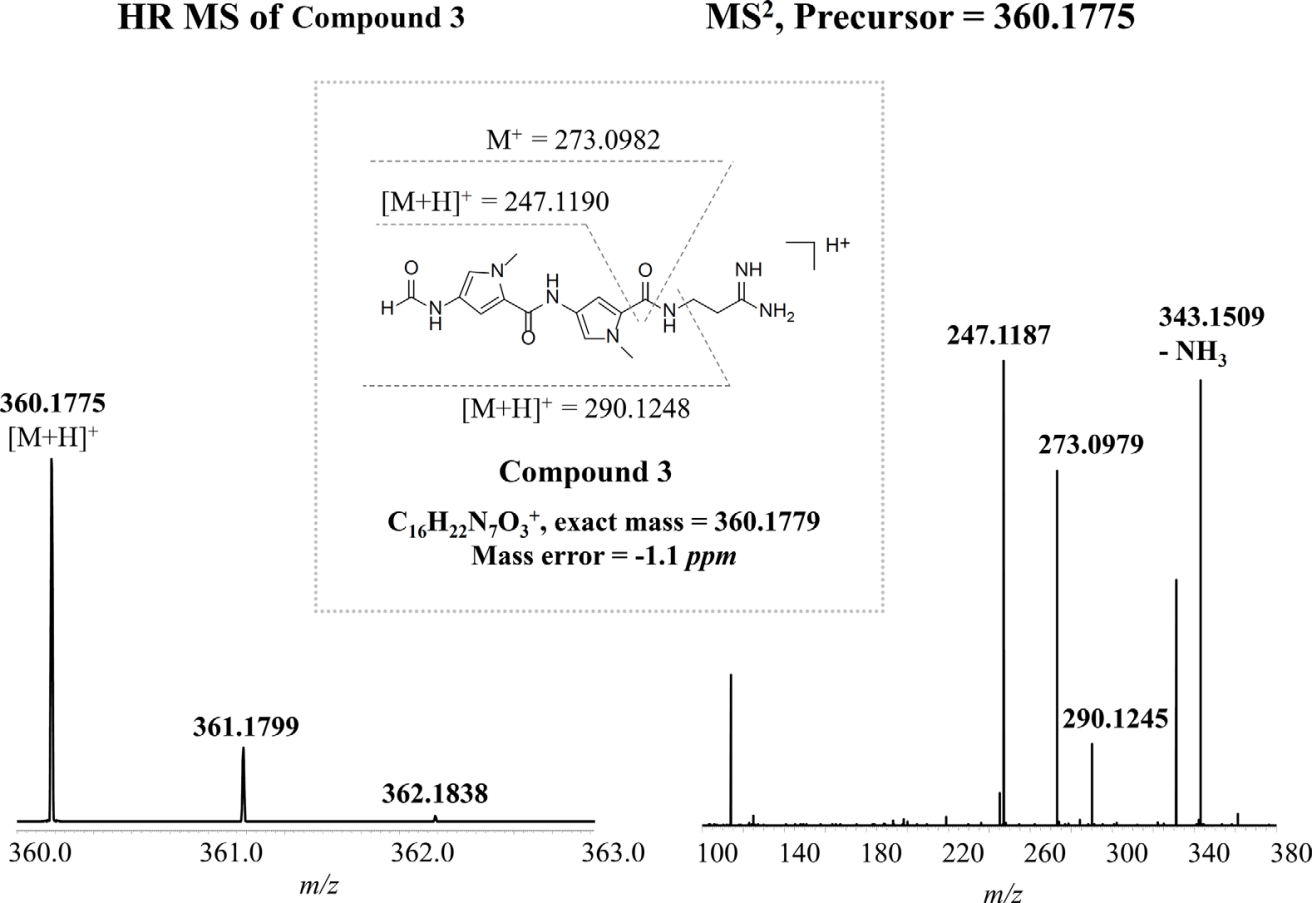
Structural elucidation of Compound 3. High resolution mass spectrum and MS/MS patterns of **3** is shown.

The discovery of two different pyrrolamides in the same producer led us to assume that there were probably more pyrrolamides, either biosynthesis intermediates or analogs, that could be produced in *S. netropsis* CGMCC 4.1650. It has been well established that various combinations of nutrient components in culture media can provoke the accumulation of diverse secondary metabolites [26]–[28]. To increase the chances of finding novel pyrrolamides in this strain, optimized media with different compositions were used to perform the fermentation trials. Then, PIS mode was utilized to search for pyrrolamides from the culture extract by monitoring ions of *m*/*z* 273 and 247, which are the two characteristic daughter ions of pyrrolamides. In this way, a putative pyrrolamide with parent *m*/*z* at 360 (compound **3**) was detected (Figure 1B). Based on the ion information of various fragments inferred from known pyrrolamide NPs, both HR-LC-ESIMS analysis and MS/MS fragmentation patterns of **3** suggested its structure as a hybrid of congocidine/distamycin, in which the guanidinoacetyl group of congocidine is replaced by a formyl group (Figure 2). The ^1^H NMR inspection further confirmed **3**′s structure (Figure S2 in File SI).

**Figure 3:**
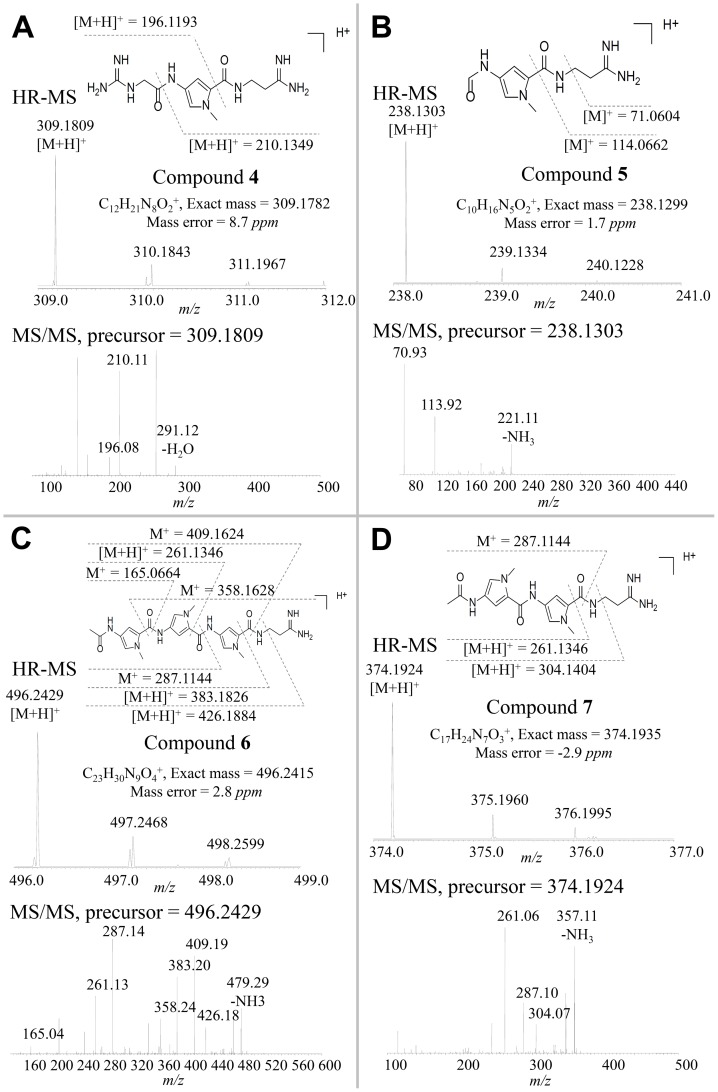
Identification of the novel pyrrolamide compounds 4 (A), 5 (B), 6 (C), and 7 (D). High resolution mass spectrum and MS/MS patterns of each compound are shown.

More intriguingly, four more minor peaks were also detected in the crude extract of *S. netropsis* fermentation culture. These peaks, with [M+H]^+^ ion at *m*/*z* 309.1809 (compound **4**), 238.1303 (compound **5**), 496.2429 (compound **6**), and 374.1924 (compound **7**), have no match to known NPs but show the same daughter ions of **1** and **2** (Figure 3). Taking into account the close retention time of these compounds to **1**, **2** and **3**, they were speculated to be novel pyrrolamides. Tandem MS analysis of these compounds confirmed the following information: (a) **4** and **5**′s structures were similar to **1** and **2**′s, respectively, but differed by consisting of a single 4-aminopyrrole-2-carboxylate unit (Figure 3A, 3B), (b) structure of **6** was as same as that of **2,** except for the guanidinoacetyl group being replaced by an acetyl group (Figure 3C), and (c) **7** differed from **6** by lacking one 4-aminopyrrole-2-carboxylate unit (Figure 3D). To our knowledge, this is the first work to report the simultaneous production of seven different pyrrolamides in the same strain.

### Characterization of Two Discrete Pyrrolamides Biosynthesis-related Gene Clusters

**Figure 4:**
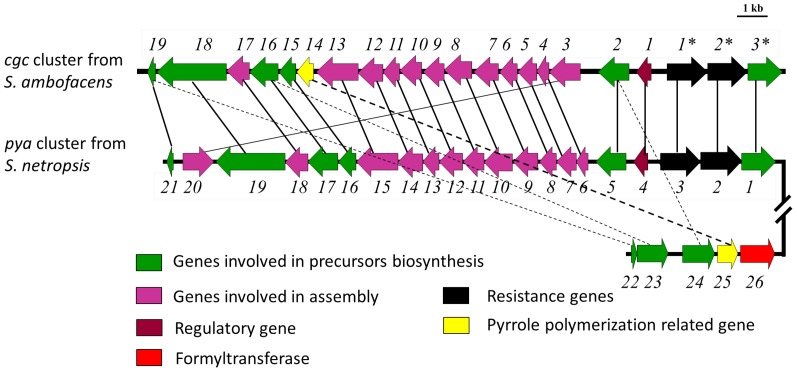
Organization of the pyrrolamides biosynthesis-related genes identified from *S. ambofaciens* (a congocidine producer) and *S. netropsis*. The deduced functions of each gene are summarized in Table S1 in File S1. Homologies in sequence are indicated by plain and dashed lines (the latter pattern is for the separate gene cluster).

**Figure 5:**
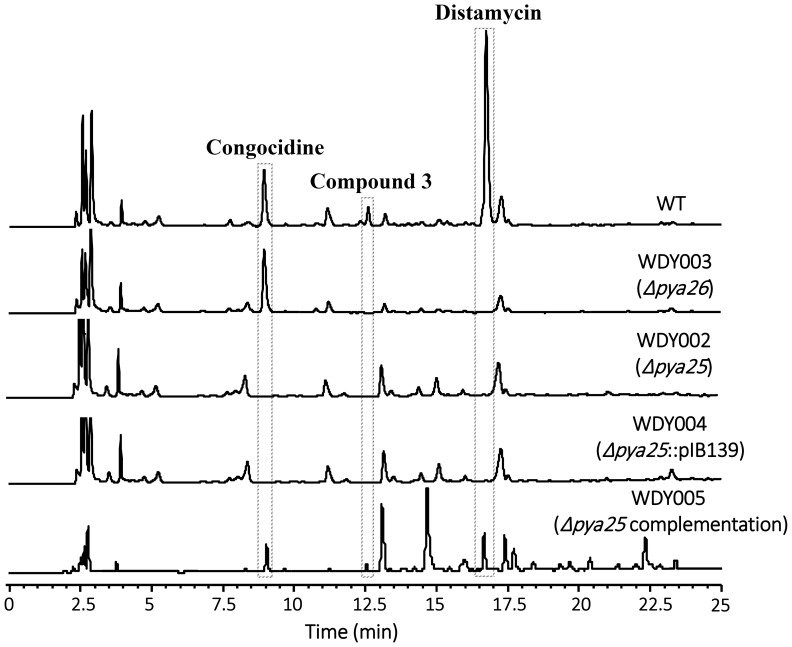
In-frame deletion of *pya25* and *pya26* in *S. netropsis*. HPLC analysis of pyrrolamides production in *S. netropsis* wild-type strain, the mutant strains WDY002 (Δ*pya25*) and WDY003 (Δ*pya26*), and the complementation strains WDY004 (negative control) and WDY005. Congocidine, Compound **3**, and Distamycin are indicated. The characteristic absorbance wave-length for pyrrolamides is 297 nm.

It is interesting to note that 4-aminopyrrole-2-carboxylate and 3-aminopropionamidine are the two common precursors for all the pyrrolamides identified in this study, implying that this group of compounds may share a common assembly pathway. To verify this hypothesis, *S. netropsis* CGMCC 4.1650 was subjected to Illumina genome sequencing. Using cgc2* as the sequence query, genome scanning of the generated 193 scaffolds, which covered 7.6 Mb of the chromosome, revealed a putative pyrrolamide biosynthetic cluster containing 21 open reading frames (ORFs) (Figure 4, *pya1* to *pya21*, Table S1 in File SI). All the homologs of congocidine biosynthetic genes can be found in this cluster except for *cgc14*, which codes for a putative amidohydrolase. This finding led us to perform another scan of the genome using *cgc14* as the query and a small gene cluster consisting of five ORFs was eventually located (Figure 4, *pya22* to *pya26*, Table S1 in File SI). Remarkably, three ORFs (*pya22* to *pya24*) within this smaller cluster encode proteins with high homology to the three free-standing NRPS domains (Cgc19, peptidyl carrier protein (PCP) domain; Cgc2 and Cgc16, condensation (C) domain) of congocidine gene cluster [24]. Of the remaining ORFs, *pya25* is the homolog of *cgc14*, and *pya26*, which encodes a putative methionyl-tRNA formyltransferase, may be responsible for the formylation of the 4-aminopyrrol group in **2** and **3**. To verify the correlation of this discrete gene cluster with the pyrrolamides production in *S. netropsis*, *pya25* and *pya26* were individually deleted in frame (Figure S3 in File SI). HPLC analysis showed that the *pya26* deletion mutant (WDY003) still produced congocidine, whereas productions of congocidine, distamycin, and **3** were all abolished in *pya25* deletion mutant (WDY002, Figure 5). To exclude the possibility that the mutagenesis could affect the transcription of the upstream or downstream ORFs, the gene *pya25* was reintroduced into the chromosome of WDY002, yielding the complementary strain WDY005. This was achieved by cloning *pya25* into the integrative vector pIB139 in which *pya25* was placed under the constitutive promoter ermE*. As the control, the empty pIB139 was introduced into WDY002, resulting in the recombinant strain WDY004. HPLC analysis of the fermentation extracts of the above mutant strains showed that WDY005 had restored the production of congocidine, distamycin, and **3** with a lower yield compared to the wild-type strain (Figure 5). These results suggested that the enzymes encoded by the additional smaller gene cluster were also involved in the pyrrolamides production. Further, *pya25*-encoded amidohydrolase may play a central role in the biosynthesis of various pyrrolamides, while Pya26 was only responsible for the formyl group containing pyrrolamides biosynthesis.

According to the heterologous expression experiment [24], the 21 genes in the main cluster, plus *pya25*, were enough for the biosynthesis of congocidine. However, the exact roles played by Pya22, Pya23 and Pya24 remain unclear. Cgc2 and Cgc16 were hypothesized by Juguet *et al*. to catalyze the sequential addition of the guanidinoacetyl-CoA and 3-aminopropionamidine through interacting with Cgc19, which carries the bipyrrole intermediate. From this point, it is intriguing to consider that *pya22*, *pya23* and *pya24* may encode another set of standalone NRPS domains, which are specifically involved in the biosynthesis of the formyl group containing pyrrolamides such as **2** and **3**. One support for this hypothesis is that these three genes and *pya26* are located in the same operon.

### An Intriguing “Iterative Strategy” may Control the Diversity of Pyrrolamide NPs

One of the most attractive questions in pyrrolamide biosynthesis is how pyrrole polymerization is controlled for incorporation into members such as **1** and **2**. In the biosynthesis of **1**, it was proposed that Cgc18 acts iteratively to load the PCP domain of itself and Cgc19 with the same pyrrole precursor, 4-acetylaminopyrrole-2-carboxylate, which is then deacetylated by Cgc14 prior to be assembled into the final skeleton [24], [25]. This model is also hypothesized to be suited to the biosynthesis of tripyrrole skeleton in **2** if the C domain of Cgc18 further catalyzes the condensation between the bipyrrole (the product of the first round of condensation) tethered with Cgc19 and the third pyrrol residue tethered with the PCP domain of Cgc18 [24]. However, this model cannot give a plausible explanation for the biosynthesis of **6** and **7**, which contain one acetyl group, since there is no ORF that encodes an acytyltransferase homolog within the cluster.

**Figure 6:**
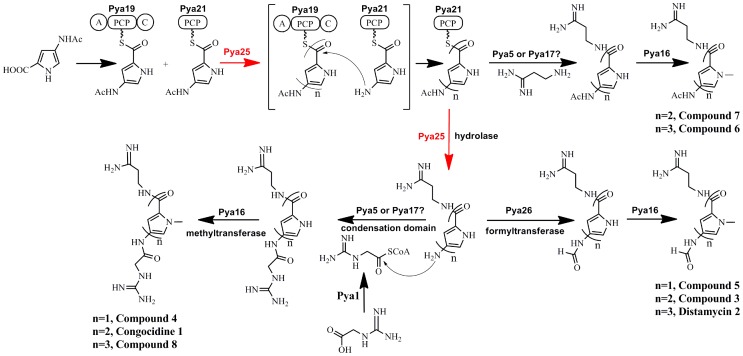
Illustration of the “iterative strategy” underlying pyrrolamide biosynthesis. The putative amidohydrolase Pya25 catalyzed the deacetylation of PCP-tethered pyrrolamide biosynthesis intermediates and determined the number of the pyrrole groups assembled into various pyrrolamides. A, adenylation domain; C, condensation domain; PCP, peptidyl carrier protein.

The above analysis inspired us to propose an “iterative strategy” underlying pyrrolamide biosynthesis in which the putative amidohydrolase Pya25 may play the central role in controlling the flow of different intermediates into the following assembly line (Figure 6). The most striking difference between our model and the reported one is that only the acetylaminopyrrole residue attached to the discrete PCP domain (Pya21) can be deacetylated. The pyrrole precursor attached to Pya19 will be kept intact before its condensation with the deacetylated aminopyrrole residue. In this way, the number of the pyrrole groups assembled into various pyrrolamides is determined by the deacetylation reaction catalyzed by Pya25 (Figure 6). Meanwhile, another free-standing condensation domain (Pya5 or Pya17), which is supposed to be responsible for the addition of 3-aminopropionamidine residue, will interact with Pya21 to catalyze the pre-release of the biosynthetic intermediates, giving rise to the production of **4**, **5**, **6** and **7**. Thus, our model fits well the biosynthesis of all the pyrrolamides discovered in this study (Figure S4 in File SI).

Polymerization of a same building block is common in NRPS-directed peptidyl NPs biosynthesis. However, few mechanisms for controlling of the block tandem number have been elucidated [29]. One example is the biosynthesis of the poly-ε-lysine (ε-PL), which is actually a cocktail of peptides containing 25–35 lysine units [30]. Hamano and colleagues have identified an unusual single-module NRPS-like membrane protein, which iteratively catalyzes the condensation of L-lysine to give products with different chain length [30]. They further demonstrate that the polymerization of L-lysine and the number of the building block incorporated are determined by the ε-PL synthetase itself rather than the ε-PL-degrading enzymes in the cell [31]. The possible mechanism is that the long tunnel or cavity formed inside the ε-PL synthetase may define the length of the final products [30]. Our work provided another example which disclosed the tricky strategy utilized by microorganism to control pyrrole polymerization in pyrrolamides biosynthesis. Pernodet and colleagues have attributed the underlying mechanism to the strict substrate specificity of the condensation domain in Cgc18 [24]. Supported by the data obtained in this study, we, on the other hand, proposed a more plausible explanation that iterative deacetylation of the pyrrole residue(s) attached to the discrete PCP domain (Pya21) specifies the various number of pyrrole rings in the final products. Though pyrrole tandem diversity can be well explained by this mechanism, a detailed *in vitro* verification of the interaction between Pya25 and other NRPS (such as Pya21) remains a subject for future work.

### Conclusions

Before this study, only seven natural pyrrolamides had been found. By applying genome scanning and precursor ion scan-directed mass spectrometry, five novel pyrrolamdies were discovered in *S. netropsis* CGMCC 4.1650, suggesting that combinatory use of different NPs mining approach possesses great potential to uncover NPs previously overlooked in routine compounds screening procedure. It maybe also intriguing to use the workflow described in this study to explore novel NPs in wider ranges, such as peptidyl NPs, since amide acid residues produced by tandem mass fragmentation can be set as monitoring ions in PIS. Moreover, we established a new model for pyrrolamide biosynthesis based on bioinformatics and mutational analysis of several key biosynthesis-related genes and interpretation of newly found compounds structures. Significantly, a deacetylation reaction catalyzed by a putative amidohydrolase was proposed to work as a switch to determine the number of pyrrole unit in various pyrrolamides. This kind of precise control of building block polymerization is an unusual example of iterative NRPS-directed peptidyl NPs biosynthesis. In conclusion, our findings not only show the practicality of scaffold-oriented discovery of potential biosynthesis intermediates and analogs, but also facilitate further engineering the biosynthetic machinery to create new classes of pyrrolamide compounds.

## Materials and Methods

### DNA Sequencing and Analysis

*S. netropsis* CGMCC 4.1650 genomic DNA was prepared through salting out method, and then sequenced by Illumina Hiseq 2000 with a 300 bp paired-end library through TruSeq method. A total of 4,176,684 paired-reads were obtained and assembled by SOAP *de novo* software (http://soap.genomics.org.cn/soapdenovo.html) with the parameters “sequence length >25 bp and base quality >20”. When the K-mer was 21, the best assembly result could be obtained. The resultant 193 scaffolds were further annotated by Glimmer software (http: //www.cbcb.umd.edu/software/glimmer/). The pyrrolamide biosynthetic gene cluster was identified from the sequenced genome via BLAST method using cgc2* as the sequence query. The pyrrolamide biosynthetic gene cluster was submitted to NCBI GenBank with the accession number KF158418.

### Degenerated PCR primers for genome scanning

Based on knowledge of the congocidine biosynthetic gene cluster and pathway, cgc2*, a pyrrolamides specific transporter protein encoding gene, was promoted as the query gene. Top eleven cgc2* homologs sharing more than 60% similarity were selected by Basic Local Alignment Search Tool (BLAST) (http://www.ncbi.nlm.nih.gov/blast/). Amino acid sequence alignments were performed with the CLUSTALW algorithm from BIOLOGYWORKBENCH 3.2 software (http://workbench.sdsc.edu/). Based on the conserved motifs of PLTSIASFW and ILDEATASI, degenerated primer pairs Cgc2-F1: CCSYTSACSTCSATCGCNTCSTTYTG and Cgc2-R1: AYSGABGCSGTBGCYTCGTCSARGA were designed by CODEHOP [32]. PCR was performed in 20 µL of volume with 5% DMSO and KOD DNA polymerase (TOYOBO). The amplification conditions for PCR were: initial denaturation at 95°C for 5 min; 30 cycles of denaturation at 95°C for 30 s, annealing at 58°C for 30 s, and extension at 68°C for 1 min; and gap infilling at 68°C for 10 min.

### Media used for pyrrolamides production

To optimize the medium for pyrrolamide production, different kinds of carbon sources, nitrogen sources, and inorganic salts were tested. Tested carbon sources were glucose, sucrose, glycerol, and soluble starch; tested nitrogen source included soybean cake, corn steep liquor, yeast extract, malt extract, Indian meal, and cottonseed meal; tested inorganic salts were NaCl, CaCO_3_, K_2_HPO_4_, MgSO_4_, FeSO_4_, and (NH_4_)_2_SO_4_. The highest production of the known pyrrolamides (distamycin and congocidine) was achieved by a medium composed of 2% glucose, 2% Indian meal, 1% CaCO_3_, 0.3% (NH_4_)_2_SO_4_, and 0.3% NaCl, at pH 7.6. It was used for novel pyrrolamide screening as well.

### Pyrrolamides extraction and purification

*S. netropsis* CGMCC 4.1650 spores were inoculated into tryptone soya broth and yeast extract medium (yeast extract 5 g/L, tryptone soya broth 30 g/L), and grown for three days at 28°C, 200 *rpm*, and then transferred into pyrrolamide fermentation medium (1∶100, volume to volume) and cultivated at 28°C, 200 *rpm* for 7 days. Mycelia were collected and re-suspended in methanol with one-tenth of the original culture volume, and then ultrasonically disrupted using KQ3200V Ultrasonic cleaning apparatus (40 kHz, 25 min). Cell pellets were eliminated by centrifugation and solvents were subsequently dried out by rotary evaporation. Culture supernatants were extracted by an equal volume of n-butanol. The organic phase was collected and evaporated to dryness. Residues from two sections were combined and re-dissolved in 1/400 of original culture volume of methanol for HPLC or LC-MS detection. Purification of pyrrolamides was performed by semi-preparative HPLC of the crude extract on a Agilent ZORBAX SB-C18 column (5 µm, 9.4×250 mm) with a flow rate of 3 mL/min over a 35 min gradient (T = 0 min, 10% B; T = 20 min, 40% B; T = 25 min, 100% B; T = 35 min, 100%; solvent A, water; solvent B, acetonitrile). The UV monitoring was set at 297 nm.

### In-frame deletion of *pya25* and *pya26*

To inactivate *pya25*, a 1918 bp upstream fragment and a 1924 bp downstream fragment were amplified from genomic DNA of *S. netropsis* by PCR using the primers PYA25_Inf_F1/PYA25_Inf_R1 and PYA25_Inf_F2/PYA25_Inf_R2, respectively (Table S2 in File SI). PCR was performed in 20 µL of volume with 5% DMSO and KOD DNA polymerase (TOYOBO). The amplification conditions were: initial denaturation at 95°C for 5 min; 30 cycles of denaturation at 95°C for 30 s, annealing at 55°C for 30 s, and extension at 68°C for 2 min; and gap infilling at 68°C for 10 min. The obtained fragments were digested with HindIII/PstI and PstI/EcoRI respectively, and cloned into the HindIII/EcoRI site of pOJ260 to give the in-frame deletion construct, which was then transferred into *S. netropsis* via *E. coli*-*Streptomyces* conjugation. Following the procedure described previously [33], the *pya25* in-frame deletion mutant strains were screened out and designated as WDY002. The same strategy was used to generate the *pya26* in-frame deletion mutant WDY003, except that the 1957 bp upstream and 1936 bp downstream fragments were amplified by PCR using primers PYA26_Inf_F1/PYA26_Inf_R1 and PYA26_Inf_F2/PYA26_Inf_R2, respectively (Table S2 in File SI). The amplification conditions for both PCR experiments were: initial denaturation at 95°C for 5 min; 30 cycles of denaturation at 95°C for 30 s, annealing at 55°C for 30 s, and extension at 68°C for 2 min; and gap infilling at 68°C for 10 min.

### Complementation of *pya25* knock out strain WDY002

To complement WDY002, a 971 bp fragment which contains the whole *pya25* gene sequence was amplified from genomic DNA of *S. netropsis* by high fidelity PCR using the primers PYA25_Com_F/PYA25_Com_R (Table S2 in File SI). The amplification conditions were: initial denaturation at 95°C for 5 min; 30 cycles of denaturation at 95°C for 30 s, annealing at 55°C for 30 s, and extension at 68°C for 1 min; and gap infilling at 68°C for 10 min. The obtained fragment was cloned into the XbaI site of pIB139, which can integrate into ФC31 phage site in *Streptomyces* chromosome. The resulting construct was then transferred into *S. netropsis* via *E. coli*-*Streptomyces* conjugation. Following the procedure described previously [33], the *Δpya25* complementation mutant strain was screened out and designated as WDY005.

### HPLC MS/MS analysis

HPLC analysis was carried out on a DIKMA Diamonsil C18 column (250×4.6 mm, 5 µm, column temperature 30°C) using an Agilent 1260 HPLC instrument. Samples were eluted with a gradient from 95∶5 A/B to 70∶30 A/B over 20 min, followed by another gradient to 40∶60 A/B over 30 min at a flow rate of 1 mL/min, and monitored at 297 nm. Twenty percent of the eluent was injected to source and eighty percent to waste. Solvent A was 0.1% formic acid in H_2_O and solvent B was 0.1% formic acid in CH_3_CN. The same column and LC gradient was used in all LC-MS analysis. High resolution MS analysis, which consisted of a full scan in positive mode followed by a data dependent fragmentation scan, was performed on a Thermo Scientific LTQ XL Orbitrap mass spectrometer equipped with a Thermo Scientific Accela 600 pump. Pyrrolamide samples extracted from the culture of *S. netropsis* CGMCC 4.1650 were used to identify the MS fragmentation fingerprint of congocidine and distamycin by selected reaction monitoring with a Hi-Hi setup (high resolution for both full scan and fragmentation scans). Daughter ions with *m/z* ratios at 273.0982 and 247.1190 were recognized to be the characteristic fragments. To screen for novel pyrrolamides, precursor ion scan was performed in positive mode on a Thermo Scientific TSQ Quantum Access MAX instrument (monitoring *m/z* at 273 and 247) equipped with a Thermo Scientific Accela 600 pump. In order to determine the elemental compositions, ions that showed distinct *m/z* ratios and absent in the medium control and an extract of the negative control strain (*Streptomyces lividans*) were selected for further analysis by high resolution MS as described above. Empirical formulae of pyrrolamides were deduced based on high resolution full scan and tandem mass spectra.

### NMR analysis

^1^H NMR spectra of compound **1** (congocidine, 2 mg), compound **2** (distamycin, 0.9 mg), and compound **3** (2 mg) were recorded on Agilent 500 MHz instrument in CD_3_OD (for compound **3**) or (CD_3_)_2_SO (for compound **1**, **2**). Compound **1**: ^1^H NMR (500 MHz, dmso) δ 10.27 (s, 1 H), 9.90 (s, 1 H), 8.70 (s, 1 H), 8.39 (s, 1 H), 8.27 (s, 1 H), 7.80 (s, 2 H), 7.18 (d, *J* = 10.1 Hz, 1 H), 6.90 (d, *J* = 5.6 Hz, 1 H), 3.96 (s, 1 H), 3.83 (s, 2 H), 3.80 (s, 1 H), 3.48 (d, *J* = 5.5 Hz, 1 H), 2.58 (t, *J* = 6.0 Hz, 1 H). Compound **2**: ^1^H NMR (500 MHz, dmso) δ 10.53 (s, 1 H), 10.10 (s, 1 H), 9.92 (d, *J* = 3.7 Hz, 1 H), 8.69 (s, 1 H), 8.43 (s, 1 H), 8.29 (s, 1 H), 8.13 (s, 1 H), 7.23 (s, 1 H), 7.20 (d, *J* = 4.3 Hz, 1 H), 7.04 (s, 1 H), 6.92 (s, 1 H), 3.84 (s, 3 H), 3.81 (s, 2 H), 3.49 (d, *J* = 5.0 Hz, 1 H), 2.59 (s, 1 H). Compound **3**: ^1^H NMR (500 MHz, cd_3_od) δ 8.53 (s, 2 H), 8.15 (s, 1 H), 7.18 (d, *J* = 1.3 Hz, 1 H), 7.15 (d, *J* = 1.0 Hz, 1 H), 6.91 (d, *J* = 1.2 Hz, 1 H), 6.89 (d, *J* = 1.3 Hz, 1 H), 3.90 (d, *J* = 8.0 Hz, 3 H), 3.66 (t, *J* = 6.6 Hz, 1 H), 2.73 (t, *J* = 6.5 Hz, 1 H).

## Supporting Information

File S1 **This file contains Figures S1 to S4 and Tables S1 to S2.**
(DOCX)

## References

[1] Cragg GM, Newman DJ (2013) Natural products: A continuing source of novel drug leads. Biochimica et Biophysica Acta 1830: 3670–3695. 23428572 10.1016/j.bbagen.2013.02.008 PMC3672862

[2] Newman DJ, Cragg GM (2012) Natural products as sources of new drugs over the 30 years from 1981 to 2010. Journal of Natural Products 75: 311–335. 22316239 10.1021/np200906s PMC3721181

[3] Baker DD, Chu M, Oza U, Rajgarhia V (2007) The value of natural products to future pharmaceutical discovery. Natural Product Reports 24: 1225–1244. 18033577 10.1039/b602241n

[4] Nett M, Ikeda H, Moore BS (2009) Genomic basis for natural product biosynthetic diversity in the actinomycetes. Natural Product Reports 26: 1362–1384. 19844637 10.1039/b817069j PMC3063060

[5] Qu XD, Lei C, Liu W (2011) Transcriptome mining of active biosynthetic pathways and their associated products in *Streptomyces flaveolus* . Angewandte Chemie-International Edition 50: 9651–9654. 21948600 10.1002/anie.201103085

[6] Kersten RD, Yang YL, Xu YQ, Cimermancic P, Nam SJ, et al (2011) A mass spectrometry-guided genome mining approach for natural product peptidogenomics. Nature Chemical Biology 7: 794–802. 21983601 10.1038/nchembio.684 PMC3258187

[7] Bumpus SB, Evans BS, Thomas PM, Ntai I, Kelleher NL (2009) A proteomics approach to discovering natural products and their biosynthetic pathways. Nature Biotechnology 27: 951–U120. 10.1038/nbt.1565 PMC2782881 19767731

[8] Wilkinson B, Micklefield J (2007) Mining and engineering natural-product biosynthetic pathways. Nature Chemical Biology 3: 379–386. 17576425 10.1038/nchembio.2007.7

[9] Menzella HG, Reeves CD (2007) Combinatorial biosynthesis for drug development. Current Opinion in Microbiology 10: 238–245. 17553731 10.1016/j.mib.2007.05.005

[10] Challis GL (2008) Mining microbial genomes for new natural products and biosynthetic pathways. Microbiology-Sgm 154: 1555–1569. 10.1099/mic.0.2008/018523-0 18524911

[11] Cane DE, Ikeda H (2012) Exploration and Mining of the Bacterial Terpenome. Accounts of Chemical Research 45: 463–472. 22039990 10.1021/ar200198d PMC3288161

[12] Corre C, Challis GL (2009) New natural product biosynthetic chemistry discovered by genome mining. Natural Product Reports 26: 977–986. 19636446 10.1039/b713024b

[13] Hertweck C (2009) Hidden biosynthetic treasures brought to light. Nature Chemical Biology 5: 450–452. 19536102 10.1038/nchembio0709-450

[14] Nguyen DD, Wu CH, Moree WJ, Lamsa A, Medema MH, et al (2013) MS/MS networking guided analysis of molecule and gene cluster families. Proceedings of the National Academy of Sciences of the United States of America 110: E2611–2620. 23798442 10.1073/pnas.1303471110 PMC3710860

[15] Neidle S (2001) DNA minor-groove recognition by small molecules. Natural Product Reports 18: 291–309. 11476483 10.1039/a705982e

[16] Asai A, Sakai Y, Ogawa H, Yamashita Y, Kakita S, et al (2000) Pyrronamycin A and B, novel antitumor antibiotics containing pyrrole-amide repeating unit, produced by *Streptomyces* sp. Journal of Antibiotics 53: 66–69. 10724011 10.7164/antibiotics.53.66

[17] Barrett MP, Gemmell CG, Suckling CJ (2013) Minor groove binders as anti-infective agents. Pharmacology & therapeutics 139: 12–23. 23507040 10.1016/j.pharmthera.2013.03.002

[18] Matteoli B, Bernardini S, Iuliano R, Parenti S, Freer G, et al (2008) In vitro antiviral activity of distamycin A against clinical isolates of herpes simplex virus 1 and 2 from transplanted patients. Intervirology 51: 166–172. 18663321 10.1159/000148199

[19] Sharma S, Doherty KM, Brosh RM Jr (2005) DNA helicases as targets for anti-cancer drugs. Current Medicinal Chemistry Anti-Cancer Agents 5: 183–199. 15992349 10.2174/1568011053765985

[20] Fuchs JE, Spitzer GM, Javed A, Biela A, Kreutz C, et al (2011) Minor groove binders and drugs targeting proteins cover complementary regions in chemical shape space. Journal of Chemical Information and Modeling 51: 2223–2232. 21819135 10.1021/ci200237c

[21] Baraldi PG, Nunez MD, Espinosa A, Romagnoli R (2004) Distamycin A as stem of DNA minor groove alkylating agents. Current Topics in Medicinal Chemistry 4: 231–239. 14754456 10.2174/1568026043451474

[22] Uria-Nickelsen M, Blodgett A, Kamp H, Eakin A, Sherer B, et al (2013) Novel DNA gyrase inhibitors: Microbiological characterisation of pyrrolamides. International Journal of Antimicrobial Agents 41: 28–35. 23142086 10.1016/j.ijantimicag.2012.08.017

[23] Iyer P, Srinivasan A, Singh SK, Mascara GP, Zayitova S, et al (2013) Synthesis and characterization of DNA minor groove binding alkylating agents. Chemical Research in Toxicology 26: 156–168. 23234400 10.1021/tx300437x PMC3618862

[24] Juguet M, Lautru S, Francou FX, Nezbedova S, Leblond P, et al (2009) An iterative nonribosomal peptide synthetase assembles the pyrrole-amide antibiotic congocidine in *Streptomyces ambofaciens* . Chemistry & Biology 16: 421–431. 19389628 10.1016/j.chembiol.2009.03.010

[25] Lautru S, Song LJ, Demange L, Lombes T, Galons H, et al (2012) A Sweet origin for the key congocidine precursor 4-acetamidopyrrole-2-carboxylate. Angewandte Chemie-International Edition 51: 7454–7458. 22707277 10.1002/anie.201201445

[26] Cordell GA, Shin YG (1999) Finding the needle in the haystack. The dereplication of natural product extracts. Pure and Applied Chemistry 71: 1089–1094.

[27] Lang G, Mayhudin NA, Mitova MI, Sun L, van der Sar S, et al (2008) Evolving trends in the dereplication of natural product extracts: New methodology for rapid, small-scale investigation of natural product extracts. Journal of Natural Products 71: 1595–1599. 18710284 10.1021/np8002222

[28] Bode HB, Bethe B, Hofs R, Zeeck A (2002) Big effects from small changes: possible ways to explore nature's chemical diversity. ChemBioChem 3: 619–627. 12324995 10.1002/1439-7633(20020703)3:7<619::AID-CBIC619>3.0.CO;2-9

[29] Hur GH, Vickery CR, Burkart MD (2012) Explorations of catalytic domains in non-ribosomal peptide synthetase enzymology. Natural Prodduct Reports 29: 1074–1098. 10.1039/c2np20025b PMC4807874 22802156

[30] Yamanaka K, Maruyama C, Takagi H, Hamano Y (2008) Epsilon-poly-L-lysine dispersity is controlled by a highly unusual nonribosomal peptide synthetase. Nature Chemical Biology 4: 766–772. 18997795 10.1038/nchembio.125

[31] Yamanaka K, Kito N, Imokawa Y, Maruyama C, Utagawa T, et al (2010) Mechanism of epsilon-poly-L-lysine production and accumulation revealed by identification and analysis of an epsilon-poly-L-lysine-degrading enzyme. Appllied and Environmental Microbiology 76: 5669–5675. 10.1128/AEM.00853-10 PMC2935060 20601519

[32] Rose TM, Henikoff JG, Henikoff S (2003) CODEHOP (COnsensus-DEgenerate hybrid oligonucleotide primer) PCR primer design. Nucleic Acids Research 31: 3763–3766. 12824413 10.1093/nar/gkg524 PMC168931

[33] Yu Y, Duan L, Zhang Q, Liao R, Ding Y, et al (2009) Nosiheptide biosynthesis featuring a unique indole side ring formation on the characteristic thiopeptide framework. ACS Chemical Biology 4: 855–864. 19678698 10.1021/cb900133x PMC2763056

