# Supplementary material for: Mining of the Pyrrolamide Antibiotics Analogs in Streptomyces netropsis Reveals the Amidohydrolase-Dependent “Iterative Strategy” Underlying the Pyrrole Polymerization
Source: PLoS One. 2014 Jun 5;9(6):e99077. doi: 10.1371/journal.pone.0099077 (PMC4047045; doi:10.1371/journal.pone.0099077)
Supplement: File S1 — This file contains Figures S1 to S4 and Tables S1 to S2. (DOCX) [file pone.0099077.s001.docx]

*Supplementary Data*

**Mining of the pyrrolamide antibiotics analogs in *Streptomyces netropsis* reveals the amidohydrolase-dependent “iterative strategy” underlying the pyrrol polymerization**

Chunlin Hao^1¶^, Sheng Huang^1¶^, Zixin Deng^1,3^, Changming Zhao^1*^, Yi Yu^1,2*^

^1^Key Laboratory of Combinatory Biosynthesis and Drug Discovery (Ministry of Education), School of Pharmaceutical Sciences, Wuhan University, Wuhan 430071, China

^2^State Key Laboratory of Bioorganic and Natural Products Chemistry, Shanghai Institute of Organic Chemistry, Chinese Academy of Sciences, 345 Lingling Road, Shanghai 200032, China

^3^Hubei Engineering Laboratory for Synthetic Microbiology, Wuhan Institute of Biotechnology, Wuhan 430075, China

¶ These authors contributed equally to this work.


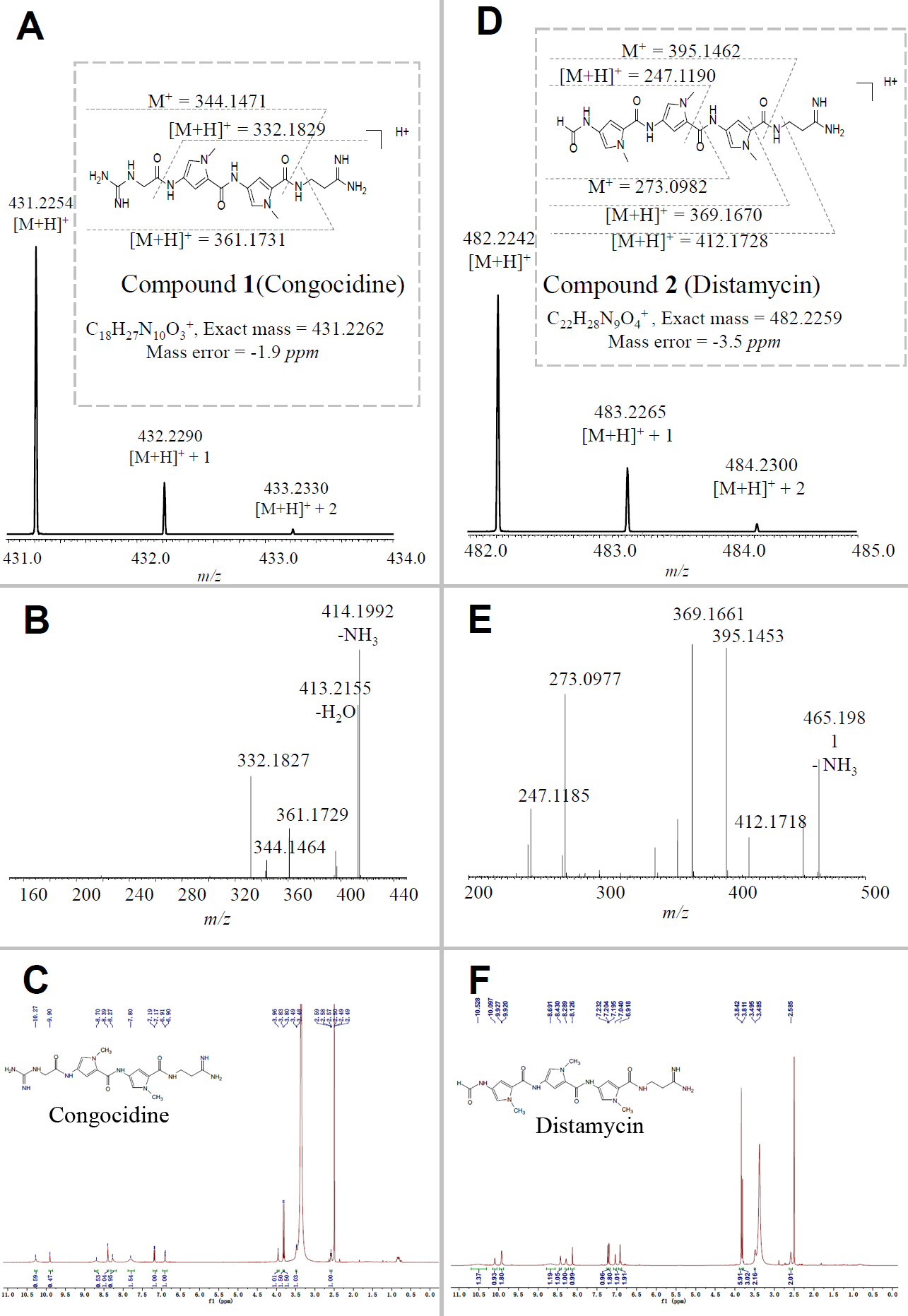


**Figure S1. MS/MS and ^1^H NMR analysis of Congocidine (1) and Distamycin (2).**


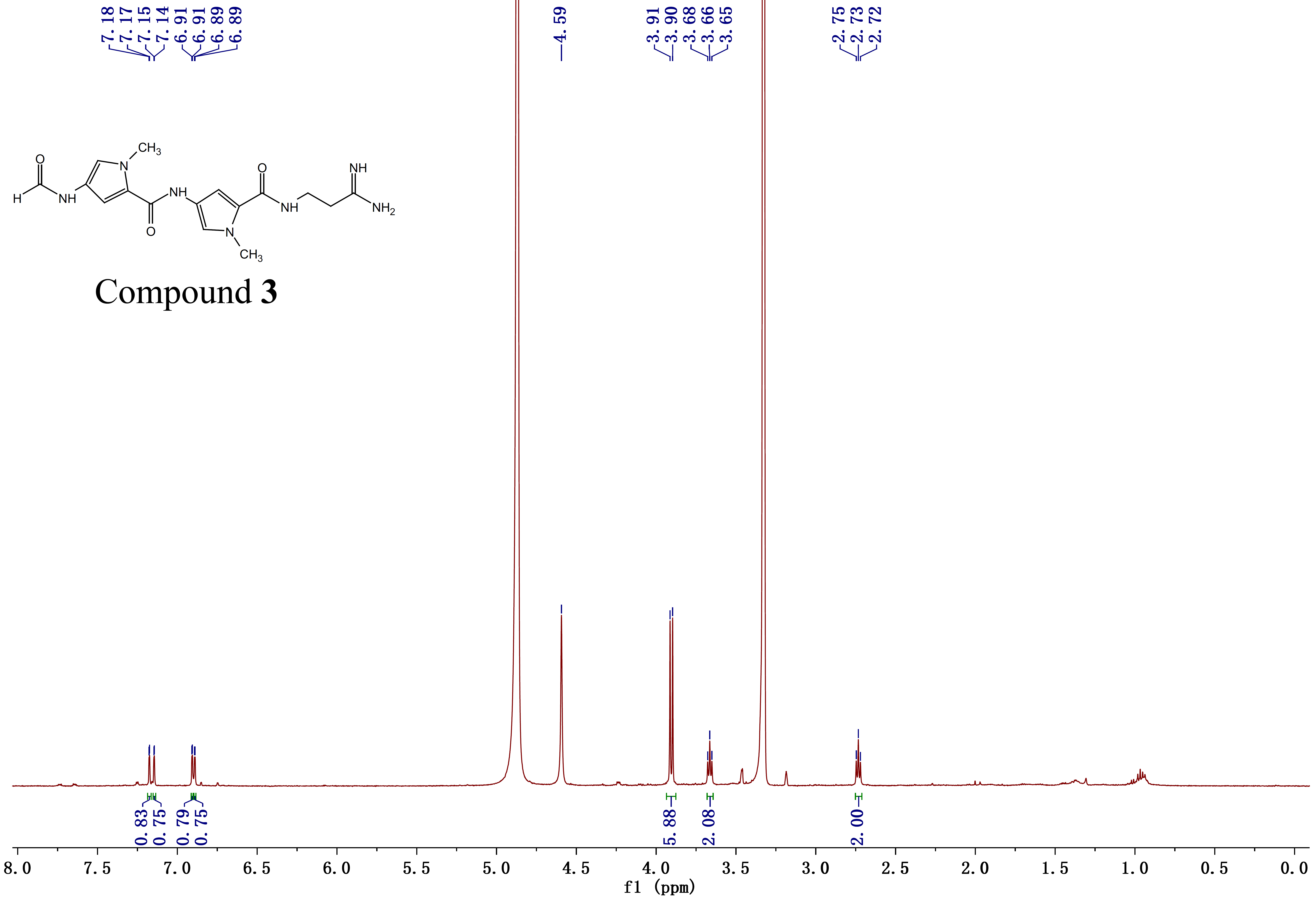


**Figure S2. ^1^H NMR spectrum of Compound 3 (500MHz in MeOH-*d*4)**.


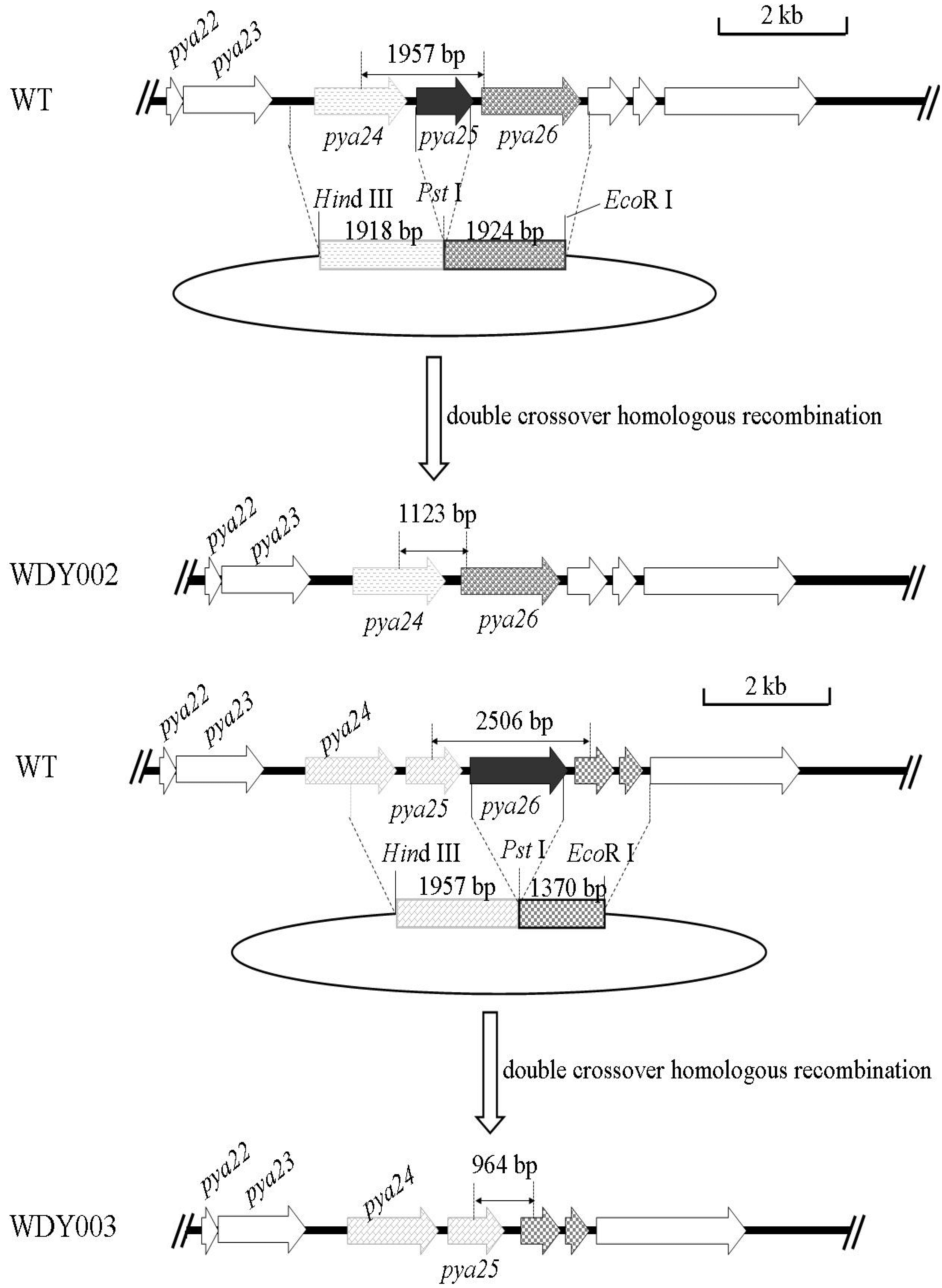


**Figure S3. Schematic representation for *pya25* and *pya26* in-frame deletion.**


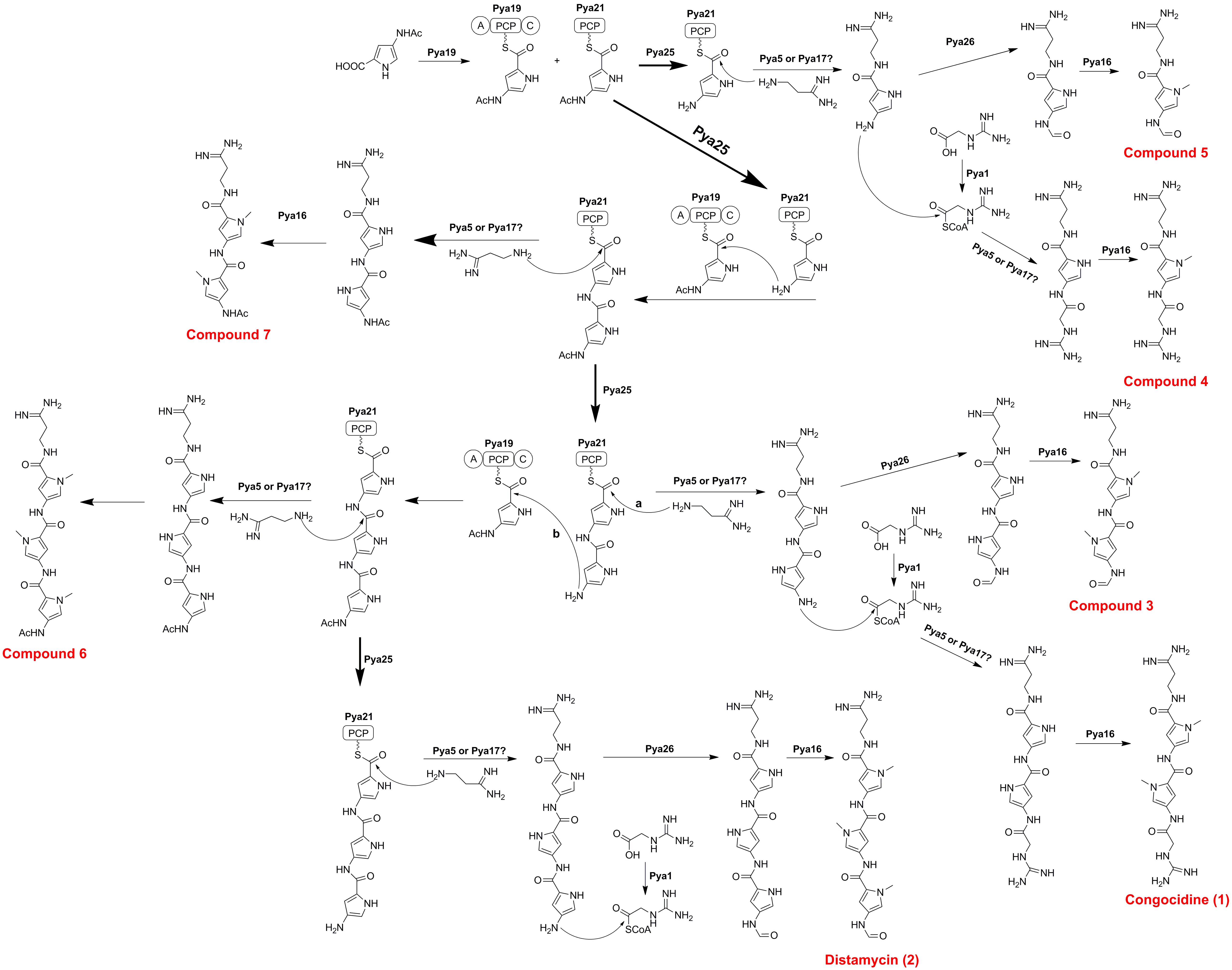


**Figure S4. Proposed biosynthetic pathway for pyrrolamide serial compounds identified in this study**

**Table S1 Deduced functions of ORFs in pyrrolamide biosynthetic gene cluster**

| **gene** | **size^a^** | **Protein homolog^b^ and origin** | **identities/positives %** | **proposed function** |
| --- | --- | --- | --- | --- |
| *pya1* | 524 | Cgc3*(CAJ88630.1), putative acyl-CoA synthetase from *Streptomyces ambofaciens* ATCC 23877 | 61/70 | acyl-CoA synthetase |
| *pya2* | 635 | Cgc2*(AAU04845.1), netropisn resistance protein submit 2 from *Streptomyces ambofaciens* ATCC 23877 | 95/96 | resistance protein |
| *Pya3* | 617 | Cgc1*(AAU04844), netropisn resistance protein submit 1 from *Streptomyces netropsis* | 95/96 | resistance protein |
| *pya4* | 225 | Cgc1(CAJ88627.1), putative two component response regulator-like from *Streptomyces ambofaciens* ATCC 23877 | 67/74 | regulator |
| *pya5* | 466 | Cgc2(CAJ86626.1), putative non-ribosomal peptide synthase from *Streptomyces ambofaciens* ATCC 23877 | 61/68 | NRPS,C domain |
| *pya6* | 182 | Cgc4(CAJ88624.1), conserved hypothetical protein from *Streptomyces ambofaciens* ATCC 23877 | 77/85 | unknown |
| *pya7* | 306 | Cgc5(CAJ88623.1), putative hihydroorotate dehydrogenase from *Streptomyces ambofaciens* ATCC 23877 | 80/85 | dehydrogenase |
| *pya8* | 266 | Cgc6(CAJ88622.1), putative creatinase from *Streptomyces ambofaciens* ATCC 23877 | 79/87 | creatinase |
| *pya9* | 378 | Cgc7(CAJ8821.1), hypothetical protein SAMR0912 from *Streptomyces ambofaciens* ATCC 23877 | 81/87 | unknown |
| *pya10* | 435 | Cgc8(CAJ88620.1), putative UDP-N-acetyl-D-mannosaminuronic acid dehydrogenase from *Streptomyces ambofaciens* | 76/86 | dehydrogenase |
| *pya11* | 327 | Cgc9(CAJ88619.1), putative epimerase from *Streptomyces ambofaciens* ATCC 23877 | 79/87 | epimerase |
| *pya12* | 358 | Cgc10(CAJ88618.1), putative glycosyl transferase from *Streptomyces ambofaciens* ATCC 23877 | 71/79 | glycosyl transferase |
| *pya13* | 252 | Cgc11(CAJ88617.1), putative nucleoside-diphosphate-sugar pyrophosphorylase from *Streptomyces ambofaciens* | 76/86 | pyrophosphorylase |
| *pya14* | 384 | Cgc12(CAJ88626.1), putative non-ribosomal synthase from *Streptomyces ambofaciens* ATCC 23877 | 74/84 | NRPS, C domain |
| *pya15* | 639 | Cgc13(CAJ88615.1), conserved hypothetical protein from *Streptomyces ambofaciens* ATCC 23877 | 74/81 | unknown |
| *pya16* | 269 | Cgc15(CAJ88613.1), putative SAM-dependent methyltransferase from *Streptomyces ambofaciens* ATCC 23877 | 76/84 | methyltransferase |
| *pya17* | 461 | Cgc16(CAJ88612.1), putative non ribosomal peptide synthetase from *Streptomyces ambofaciens* ATCC 23877 | 70/78 | NRPS |
| *pya18* | 349 | Cgc17(CAJ88611.1), putative dehydrogenase from *Streptomyces ambofaciens* ATCC 23877 | 79/85 | dehydrogenase |
| *pya19* | 1064 | Cgc18(CAJ88610.1), putative non-ribosomal peptide synthetase from *Streptomyces ambofaciens* ATCC 23877 | 63/72 | NRPS, A-PCP-C domain |
| *pya20* | 476 | Cgc3(CAJ88625.1), putative aldehyde dehydrogenase from *Streptomyces ambofaciens* ATCC 23877 | 60/71 | dehydrogenase |
| *pya21* | 96 | Cgc19(CAJ88609.1), putative nonribosomal peptide synthetase from *Streptomyces ambofaciens* ATCC 23877 | 74/83 | NRPS, PCP domain |
| *pya22* | 90 | Cgc19(CAJ88609.1), putative nonribosomal peptide synthetase from *Streptomyces ambofaciens* ATCC 23877 | 76/82 | NRPS, PCP domain |
| *pya23* | 474 | Cgc2(CAJ88626.1), putative non-ribosomal peptide synthase from *Streptomyces ambofaciens* ATCC 23877 | 58/6 | NRPS, C domain |
| *pya24* | 490 | Cgc16(CAJ88612.1), putative non ribosomal peptide synthetase from *Streptomyces ambofaciens* ATCC 23877 | 51/61 | NRPS, C domain |
| *pya25* | 304 | Cgc14(CAJ88614.1), putative metal-dependent hydrolase of the TIM-barel fold from *Streptomyces ambofaciens* | 74/82 | hydrolase |
| *Pya26* | 526 | WP_005464514.1, methionyl-tRNA formyltransferase from *Saccharomonospora glauca* | 45/60 | formyltransferase |

a Numbers are in amino acids.

b NCBI accession numbers are given in parentheses.

**Table S2. PCR primers used in this study**

| Primer | Sequence* | Function |
| --- | --- | --- |
| Cgc2-F1 | CCSYTSACSTCSATCGCNTCSTTYTG | Genome mining |
| Cgc2-R1 | AYSGABGCSGTBGCYTCGTCSARGA | Genome mining |
| PYA25_Inf_F1 | TTTAAGCTTAAAGCCCGGAGAAACGATGA | *pya25* in-frame deletion |
| PYA25_Inf_R1 | AAACTGCAGAGAAGTGACGGTAGTACTCG | *pya25* in-frame deletion |
| PYA25_Inf_F2 | AAACTGCAGCGTCCAGTCCGCGAGAGGAGCA | *pya25* in-frame deletion |
| PYA25_Inf_R2 | TTTGAATTCCGCGTCGGCCGTGGAGTTCG | *pya25* in-frame deletion |
| PYA26_Inf_F1 | TTTAAGCTTGCCGTACCGCTGGCCATCACCATC | *pya26* in-frame deletion;  WDY002 verification |
| PYA26_Inf_R1 | AAACTGCAGCCTTGTCACGGGGTCAGCACC | *pya26* in-frame deletion;  WDY002 verification |
| PYA26_Inf_F2 | AAACTGCAGTCTACGAATCGGAGGGAATA | *pya26* in-frame deletion |
| PYA26_Inf_R2 | TTTGAATTCTGTAGTGGTCGTGCTCGAAC | *pya26* in-frame deletion |
| PYA26-id-F | ACGGCGCAGGAGCTGAACAT | WDY003 verification |
| PYA26-id-R | TGGGCTCGAAGCTGAAGAAG | WDY003 verification |
| PYA25_Com_F | AAATCTAGAGACAACCGAAAGGGGTCCCT | *Δpya25* complementation |
| PYA25_Com_R | TTTACTAGTTGTCACCGGTGCTCCTCTCG | *Δpya25* complementation |

*Restriction sites for HindIII (AAGCTT), EcoRI (GAATTC), PstI (CTGCAG), XbaI (TCTAGA), and SpeI (ACTAGT) are underlined
